# Towards precision cardiometabolic prevention: results from a machine learning, semi-supervised clustering approach in the nationwide population-based ORISCAV-LUX 2 study

**DOI:** 10.1038/s41598-021-95487-5

**Published:** 2021-08-06

**Authors:** Guy Fagherazzi, Lu Zhang, Gloria Aguayo, Jessica Pastore, Catherine Goetzinger, Aurélie Fischer, Laurent Malisoux, Hanen Samouda, Torsten Bohn, Maria Ruiz-Castell, Laetitia Huiart

**Affiliations:** 1 Deep Digital Phenotyping Research Unit, Department of Population Health, Luxembourg Institute of Health, 1A-B, rue Thomas Edison, 1445 Strassen, Luxembourg; 2 Center of Epidemiology and Population Health UMR 1018, Inserm, Gustave Roussy Institute, Paris South - Paris Saclay University, Villejuif, France; 3 Quantitative Biology Unit, Luxembourg Institute of Health, 1A-B, rue Thomas Edison, 1445 Strassen, Luxembourg; 4 University of Luxembourg, 2, avenue de l’Université, 4365 Esch-sur-Alzette, Luxembourg

**Keywords:** Biomarkers, Cardiology, Diseases, Endocrinology, Medical research, Risk factors

## Abstract

Given the rapid increase in the incidence of cardiometabolic conditions, there is an urgent need for better approaches to prevent as many cases as possible and move from a one-size-fits-all approach to a precision cardiometabolic prevention strategy in the general population. We used data from ORISCAV-LUX 2, a nationwide, cross-sectional, population-based study. On the 1356 participants, we used a machine learning semi-supervised cluster method guided by body mass index (BMI) and glycated hemoglobin (HbA1c), and a set of 29 cardiometabolic variables, to identify subgroups of interest for cardiometabolic health. Cluster stability was assessed with the Jaccard similarity index. We have observed 4 clusters with a very high stability (ranging between 92 and 100%). Based on distinctive features that deviate from the overall population distribution, we have labeled Cluster 1 (N = 729, 53.76%) as “Healthy”, Cluster 2 (N = 508, 37.46%) as “Family history—Overweight—High Cholesterol “, Cluster 3 (N = 91, 6.71%) as “Severe Obesity—Prediabetes—Inflammation” and Cluster 4 (N = 28, 2.06%) as “Diabetes—Hypertension—Poor CV Health”. Our work provides an in-depth characterization and thus, a better understanding of cardiometabolic health in the general population. Our data suggest that such a clustering approach could now be used to define more targeted and tailored strategies for the prevention of cardiometabolic diseases at a population level. This study provides a first step towards precision cardiometabolic prevention and should be externally validated in other contexts.

## Introduction

Globally, the epidemic of cardiometabolic diseases, such as type 2 diabetes and hypertension, is rising, thus there is an urgent need for better tools to manage the crisis and prevent as many cases as possible^1^. Primary prevention has been shown to be possible; lifestyle intervention, medication or bariatric surgery strategies have shown to be efficient to reduce the incidence of type 2 diabetes or hypertension in at-risk individuals^2–4^. However, these strategies may be sub-optimal and do not rely on a complete understanding of the detailed cardiometabolic profiles of the general population^5^. Most of the screening and prevention strategies are simply based on a few factors such as age, body mass index, metabolic syndrome, hyperglycemia or risk score such as Findrisc^6^ to identify eligible people. We tend to omit a potentially high variability in individuals at a given level of risk, for instance in terms of genetic profiles^7^, inflammation, oxidative stress^8^, insulin resistance^9^ and hepatic gluconeogenesis^10^, that could open a window of opportunity for more relevant strategies.

Cluster analyses are useful approaches to identify subgroups with different cardiometabolic profiles. Such an approach has recently been developed among people with diabetes, the analysis revealing 5 subgroups with different clinical profiles and risks of diabetes-related complications^11^, but has never been investigated in the general population at large scale^12^. Besides, clustering approaches used in the litterature so far were mostly unsupervised where it is assumed that there is no outcome variable nor is anything known about the relationships between the observations in the data set, which is not a reliable hypothesis with respect to cardiometabolic prevention. Semi-supervised clustering techniques may therefore be more adapted to derive meaningful groups^13^, similarly to what has been recently suggested in people with type 1 diabetes^14^, to redefine the way we consider, prevent and treat cardiometabolic diseases in the general population, not as independent entities but rather with a more comprehensive, patient-centered, approach.

Therefore, based on the unique set of cardiometabolic data available in the nationwide population-based ORISCAV-LUX 2 study, our objective was to stratify the general population in terms of cardiometabolic profiles with a high level of granularity, guided by two key factors to assess cardiometabolic health, namely (1) body mass index (BMI), the most frequently used indicator to evaluate adiposity in large populations and an established risk factor of numerous cardiometabolic disorders, highly correlated with various cardiometabolic and cardiovascular risk factor and (2) glycated hemoglobin (HbA1c), a reliable and documented biomarker of glycemic control that is also correlated with many cardiometabolic conditions and surrogate markers^15–18^. This new clustering will help to have a better understanding of the cardiometabolic health of the general population and might eventually help to tailor and target early prevention strategies to people who would benefit the most, thereby representing a first step towards precision prevention for cardiometabolic diseases.

## Materials and methods

### ORISCAV-LUX 2 study

The “Observation of cardiovascular risk factors in Luxembourg” (ORISCAV-LUX) 2 is the second wave of the nationwide cross-sectional, population-based ORISCAV-LUX study. The ORISCAV-LUX 1 survey, conducted between November 2007 and January 2009, was the first nationwide cross-sectional survey of cardiovascular health monitoring in Luxembourg with the objective of describing baseline information on the prevalence of “traditional” cardiovascular risk factors, including obesity, hypertension, diabetes mellitus, lipid disorders, smoking and physical inactivity among the general adult population in Luxembourg^19^. The second wave of ORISCAV-LUX was initiated in 2016 to update and monitor the evolution of cardiometabolic parameters in the general population. An extended set of health indicators, new clinical examinations and self-reported information were then integrated in this second round of data collection. The data collection workflow has already been detailed extensively elsewhere^20^. Informed consent was obtained from all participants. The study design and information collected were approved by the National Research Ethics Committee (CNER, No 201,505/12) and the National Commission for Private Data Protection (CNPD). All methods were carried out in accordance with the Declaration of Helsinki, 2008.

### Study population

**Figure 1:**
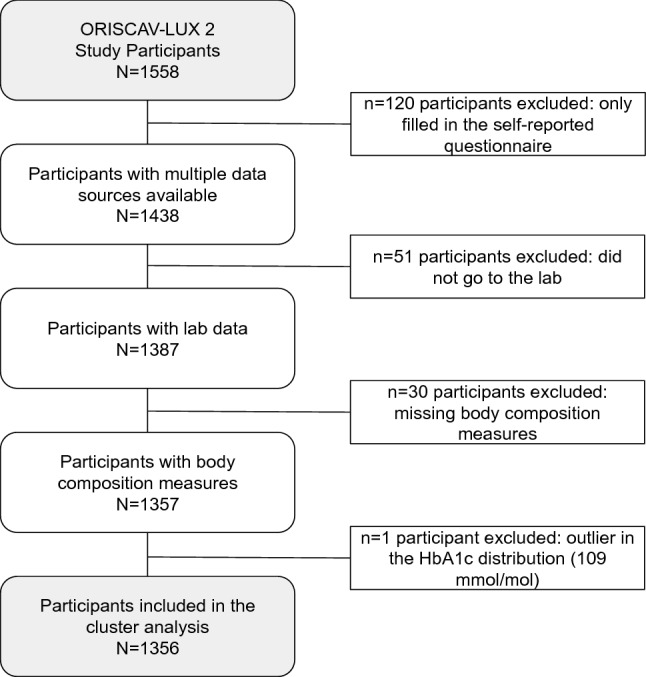
Flow-chart. Total participants included in the study (ORISCAV-LUX 2 study, N = 1356).

We included participants from the second wave of the ORISCAV-LUX study (2016–2018), where more detailed information on cardiometabolic health was available. We initially included 1558 participants, then excluded participants who only filled in the self-administrated questionnaire (n = 120), did not get a lab test (n = 51), with no body composition measures available (n = 30) and an outlier in the HbA1c distribution (HbA1c = 109 mmol/mol, n = 1). Therefore, we finally considered N = 1356 participants in the present analysis (see flow chart, Fig. 1).

### Clinical and laboratory data assessment

HbA1c was measured on an HPLC analyser, Tosof G8™. Heart rate, pulse wave velocity, central pressure, arterial age, lying position blood pressure were measured with Complior™. ECG were read and interpreted by a cardiologist and then categorized as normal or abnormal. Bioimpedanciometry measures of body fat percentage in the trunk, muscle mass in the trunk, total fat and fat free mass in the trunk were assessed with a Tanita™ digital scale. Insulin was measured on Abbott immunology analyser (chemiluminescence technique). Insulin resistance was assessed with the HOMA-IR index, calculated as Insulin (mIU/l) × Glucose (mmol/l)/22.5. Insulin sensitivity was estimated with the Quicki index, calculated as 1/[log (Insulin, mUI/l) + log (Glucose, mg/dl)]. Glomerular filtration Rate was estimated with the MDRD formula.

### Cluster analysis

We performed a semi-supervised cluster analysis guided by BMI and HbA1c to identify subgroups of interest^13^. Five measures, i.e. the means and variances of BMI and HbA1c, as well as the covariance between BMI and Hba1c, were predicted for each individual using reinforcement learning trees (RLT), a type of tree-based machine learning technique^21^. The five clustering variables (RLT-predicted means and variances of BMI and HbA1c and their covariance) were standardized and a k-means clustering algorithm^22^ with Euclidean distance was applied. Clustering was tested with and without taking the covariance between BMI and HbA1C into account.

A set of 51 cardiometabolic factors was available in ORISCAV-LUX 2. The factors of body fat and muscle mass from different body parts were highly correlated (pearson coefficient > 0.95), so we only kept the body fat and muscle mass from the trunk for further analysis to increase clustering stability. Overall, we used a subset of 31 factors to be included in the cluster analysis (the remaining factors were only used a posteriori for illustrative purposes, see Table 1). RLT prediction was performed based on the following set of cardiometabolic factors: demographic (age and sex), clinical (ECG interpretation, heart rate, carotid-femoral pulse wave velocity, central pressure, arterial age, defined as the average age for a given carotid-femoral pulse wave velocity^23^, lying position blood pressure), anthropometric (waist circumference, hip circumference, thigh circumference, waist to hip ratio, anthropometrically predicted visceral adiposity^22^, body fat percentage in the trunk, muscle mass in the trunk, total fat and fat free mass in the trunk), and laboratory (insulin, insulin resistance, insulin sensitivity, glomerular filtration rate, creatinine, total cholesterol, LDL cholesterol, HDL-cholesterol, triglycerides, CRP) measures. A missing at random mechanism was assumed and missing values were imputed using multiple imputation by chained equations (mice R package^24^).

**Table 1:** Study characteristics in the overall population. (ORISCAV-LUX 2 study, N = 1356).

Label	Overall population
N (%)	1356 (100%)
Variables	Median [1st–3rd Quartiles] or n [%]
**Sociodemographic, lifestyle and other health factors**	
Sex (female, %)*	709 [52.29%]
Age (years)*	51.11 [41.94–60.15]
Equivalised disposable income (€/month)	3571.43 [2625.00–5000.00]
Sedentary occupation (% yes)	755 [55.68%]
Total physical activity (MET-minute/week)	3492.00 [1779.75–6084.00]
Time spent sitting (mn/day)	360.00 [210.00–480.00]
Smoking status (never smoker, %)	806 [59.44%]
Vigorous physical activity (MET-minute/week)	960.00 [0.00–2880.00]
Moderate physical activity (MET-minute/week)	720.00 [200.00–1680.00]
Walking (MET-minute/week)	990.00 [396.00–2079.00]
Personal history of cancer (%yes)	54 [3.98%]
Self-perceived health (1 = excellent, 5 = poor)	3.00 [2.00–3.00]
**Diabetes-related factors**	
HbA1c (mmol/mol)	36.00 [33.00–39.00]
Fasting blood glucose (mg/dl)*	89.00 [83.00–96.00]
HOMA-IR Index*	1.56 [1.10–2.34]
Insulin (μIU/ml)*	7.10 [5.10–9.90]
Quicki—insulin sensitivity index*	0.36 [0.34–0.38]
Family history of diabetes (%yes)	296 [21.83%]
Personal history of diabetes (%yes)	72 [5.31%]
**Cardiovascular health**	
Vascular age (years)*	47.00 [37.00–58.00]
Central pulse pressure (mmHG)*	39.00 [33.00–48.00]
Carotid-femoral pulse wave velocity (m/s)*	7.90 [6.90–9.20]
Resting heart rate (bpm)*	58.00 [52.00–64.00]
ECG reading (abnormal, %)*	192 [14.16%]
Systolic blood pressure in lying position (mmHg)*	124.50 [114.50–135.50]
Diastolic blood pressure in lying position (mmHg)*	77.00 [71.00–83.50]
Family history of hypertension (%yes)	575 [42.40%]
Personal history of hypertension (%yes)	468 [34.51%]
Family history of stroke before the age of 45 (%yes)	29 [2.14%]
Family history of myocardial infarction (%yes)	144 [10.62%]
**Lipids and biomarkers**	
Total cholesterol (mg/dl)*	202.00 [179.00–229.00]
HDL cholesterol (mg/dl)*	56.00 [47.00–66.00]
LDL cholesterol (mg/dl)*	124.00 [102.20–148.00]
Triglycerides (mg/dl)*	88.00 [66.00–123.00]
GFR—MDRD formula (ml/min/1.73 m^2^)*	83.31 [75.29–92.21]
CRP (mg/l)*	1.14 [1.00–2.39]
Family history of high cholesterol (%yes)	561 [41.37%]
Creatinine (mg/dl)*	0.82 [0.75–0.93]
Body mass index (kg/m^2^)	25.45 [22.94–28.78]
Waist to hip ratio*	0.89 [0.82–0.95]
Thigh circumference (cm)*	57.90 [54.40–62.00]
Hip circumference (cm)*	100.10 [95.20–106.20]
Waist circumference (cm)*	88.70 [80.60–98.00]
**Anthropometry**	
Anthropometrically predicted visceral adiposity (cm^2^)*	8.00 [5.00–11.00]
Total fat mass percentage (%)	27.90 [22.20–34.40]
Total fat mass (kg)	20.50 [15.47–26.42]
Total free fat mass (kg)	51.90 [44.08–64.00]
Fat mass in the left arm (kg)	1.00 [0.70–1.40]
Fat mass percentage in the left arm (%)	25.20 [19.50–33.30]
Fat free mass in the left arm (kg)	2.80 [2.20–3.70]
Predicted muscle mass in the left arm (kg)	2.60 [2.10–3.50]
Fat mass in the left leg (kg)	3.60 [2.60–4.70]
Fat mass percentage in the left leg (%)	29.40 [20.28–38.50]
Fat free mass in the left leg (kg)	8.60 [7.20–10.60]
Predicted muscle mass in the left leg (kg)	8.10 [6.80–10.10]
Fat mass in the right arm (kg)	0.90 [0.70–1.30]
Fat mass percentage in the right arm (%)	24.20 [18.60–32.30]
Fat free mass in the right arm (kg)	2.80 [2.20–3.70]
Predicted muscle mass in the right arm (kg)	2.60 [2.10–3.50]
Fat mass in the right leg (kg)	3.60 [2.60–4.80]
Fat mass percentage in the right leg (%)	29.95 [20.30–38.32]
Fat free mass in the right leg (kg)	8.60 [7.30–10.80]
Predicted muscle mass in the right leg (kg)	8.20 [6.90–10.20]
Fat mass in the trunk (kg)*	11.50 [8.20–15.03]
Fat mass percentage in the trunk (%)*	28.20 [22.10–33.60]
Fat free mass in the trunk (kg)*	29.00 [25.00–35.20]
Predicted muscle mass in the trunk (kg)*	27.80 [23.98–33.90]

*Features used in the cluster analysis.

Clustering stability was assessed using clusterboot function from the fpc R package. The data is resampled 100 times using bootstrap and the Jaccard similarities^25^ of the original clusters to the most similar clusters in the resampled data are computed. The mean over these similarities is used as an index of the stability of a cluster. The assessment was applied to the clustering with the number of clusters from 3 and 8. We chose the clustering with the highest mean Jaccard similarity index of the clusters and the smallest cluster greater than 20 participants. Clusters were ordered by increasing HbA1c median. Each cluster was then described according to the variables used for the clustering, but also with additional illustrative variables: lifestyle factors (physical activity assessed with the International Physical Activity (IPAQ) questionnaire, time spent in seated position and smoking status categorized into never, former and current smoker), equivalised disposable income, sedentary occupation and other health factors such as self-perceived health (five categories from excellent to poor), family history of diabetes, hypertension, hypercholesterolemia and personal history of diabetes, cancer and hypertension.

**Figure 2:**
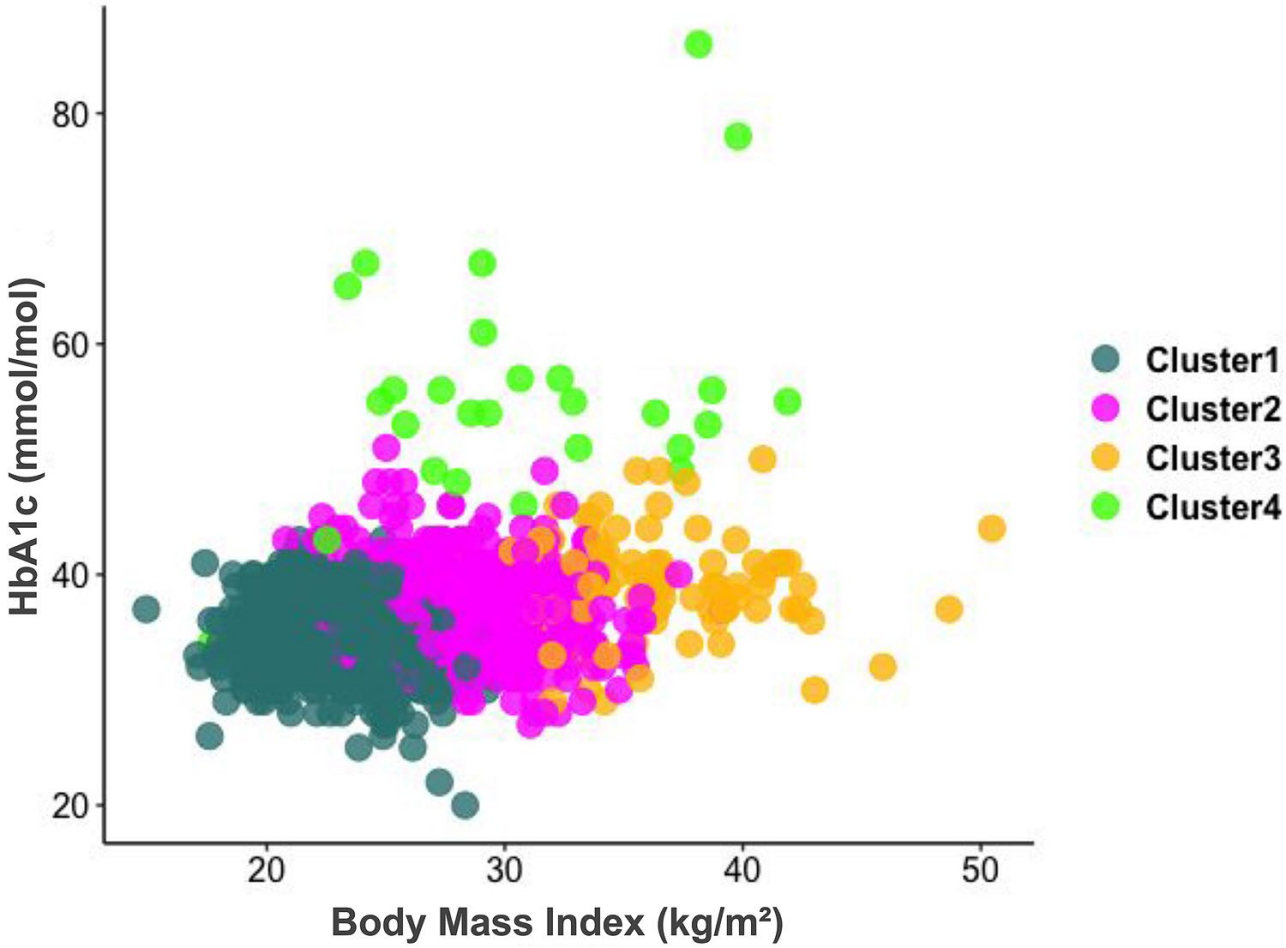
Scatter plot of body mass index and HbA1c distribution (ORISCAV-LUX 2 study, N = 1356).

**Figure 3:**
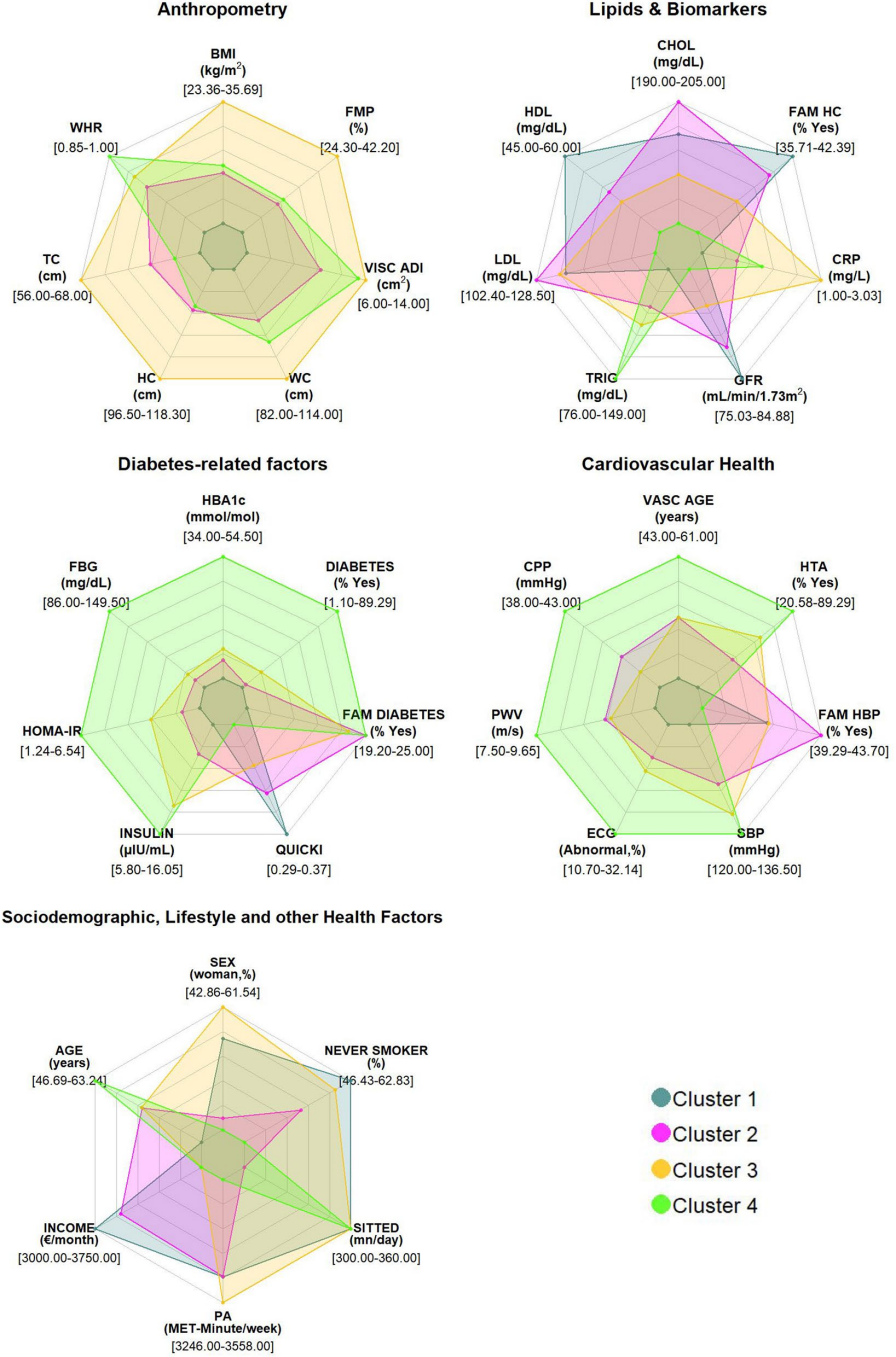
Radar diagrams of the median values for each cluster, according to 35 key diabetes-related factors, anthropometry, lipids and biomarkers, cardiovascular health, sociodemographic, lifestyle and other health factors. *BMI* body mass index, *FMP* fat mass percentage, *VISC ADI* anthropometrically predicted visceral adiposity, *WC* waist circumference, *HC* hip circumference, *TC* thigh circumference, *WHR* waist-to-hip ratio, *CHOL* total cholesterol, *FAM HC* family history of hypercholesterolemia, *CRP* C-reactive protein, *GFR* glomerular filtration rate, *TRIG* triglycerides, *LDL* LDL cholesterol, *HDL* HDL cholesterol, *HbA1c* glycated hemoglobin, *DIABETES* diabetes diagnosis, *FAM DIABETES* family history of diabetes, *QUICKI* quantitative insulin sensitivity check index, *INSULIN* insulin, *HOMA-IR* homeostatic model assessment for insulin resistance, *FBG* fasting blood glucose, *VASC AGE* vascular age, *HTA* hypertension diagnosis, *FAM HBP* family history of high blood pressure, *SBP* systolic blood pressure, *ECG* electrocardiogram, *PWV* pulse wave velocity, *CPP* central pulse pressure, *SEX* sex, *NEVER SMOKER* never smoker, SITTING time spent sitting, *PA* physical activity, *INCOME* income, *AGE* age. For each feature, we computed the relative difference, expressed in percentage, between the median value (or frequency for categorical variables) in the cluster and the median value (or frequency for categorical variables) in the overall population.

Data are presented in Table 1 as n [%] and median [min, max] for categorical and continuous variables, respectively in the entire population In Table 2, study participants’ characteristics are displayed according to their clusters. In Table 2, we also computed the average 10-year cardiovascular risk [%] per cluster, based on either the SCORE^26^ (validated for people < 70 years and no previous cardiovascular disease or type 2 diabetes mellitus) or the ADVANCE^27^ (validated for people with type 2 diabetes) risk score, whichever was most appropriate. We used the median values of the continuous variables, and considered that the binary variables were present if more than 50% of the cluster were concerned. In Fig. 2, a scatter plot of body mass index and HbA1c distribution was computed and stratified by cluster group. In Fig. 3, we have plotted the distribution of the clusters in radar diagrams according to 35 key characteristics grouped in 5 themes (Diabetes-related factors, Anthropometry, Lipids & Biomarkers, Cardiovascular Health, Sociodemographic, Lifestyle and other Health Factors). For each feature, we computed the relative difference, expressed in percentage, between the median value (or frequency for categorical variables) in the cluster and the median value (or frequency for categorical variables) in the overall population.

**Table 2:**
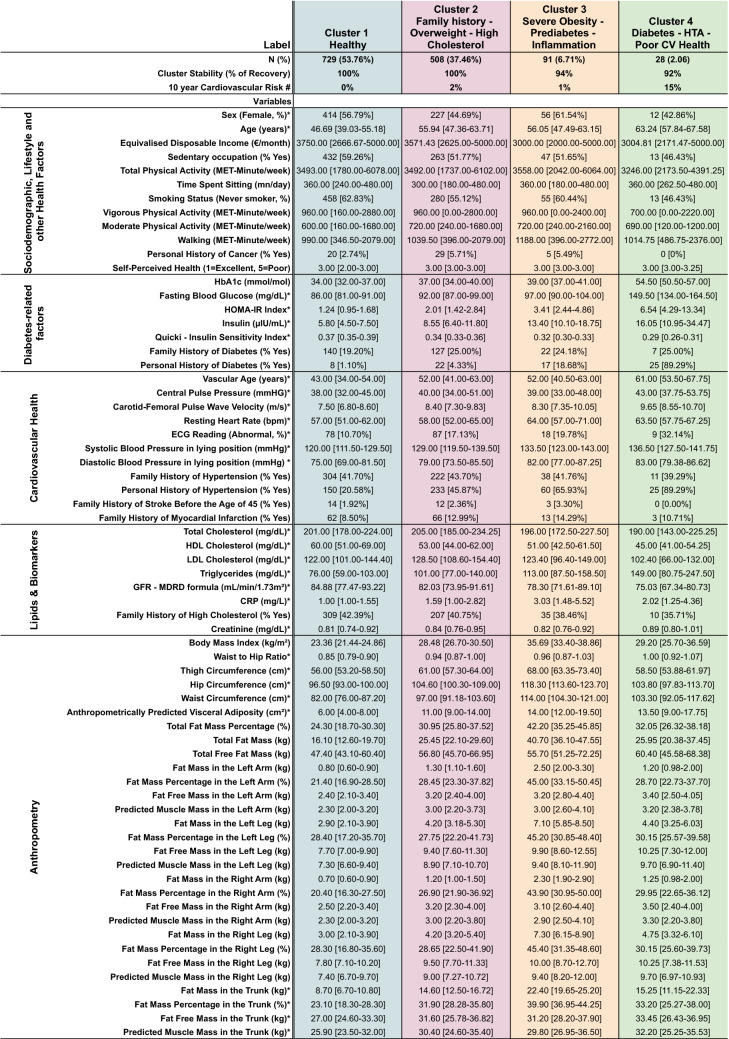
Study characteristics by cluster. (ORISCAV-LUX 2 study, N = 1356).

*Features used in the cluster analysis.

^#^Estimated with the SCORE risk score for Clusters 1-3 and with the ADVANCE risk score for Cluster 4.

## Results

### Population study characteristics

The RLT model without taking the covariance between BMI and HbA1C into account provided the most stable clusters. We tested iteratively clustering with k = 3 to 8 and we defined the final number of clusters as the one which maximized the stability index while ensuring a sufficient number of individuals in each group, with at least 20 individuals. Therefore, the optimal number of clusters appeared to be 4 and the analysis revealed a very high level of stability, with Jaccard similarity index values of 100%, 100%, 94% and 92% for clusters 1, 2, 3 and 4 respectively (Table 1). Based on the extensive description of characteristics of individuals in each cluster, Cluster 1 was labeled “Healthy”, Cluster 2 was labeled “Family history—Overweight—High Cholesterol”, Cluster 3 was labeled “Severe Obesity—Prediabetes—Inflammation” and Cluster 4 was labeled “Diabetes—Hypertension—Poor CV Health”.

Cluster 1 “Healthy” encompassed a total of N = 729 participants (53.76% of the total population). Compared to the overall population (Table 1), members of Cluster 1 were characterized by young individuals (median, m = 46.69 years old) with a low median HbA1c level (m = 34.00 mmol/mol) and low BMI (m = 23.36 kg/m^2^) (Fig. 2). They also had the lowest values for anthropometric features such as waist-to-hip ratio (m = 0.85), fat mass percentage (m = 24.30%) or predicted visceral adiposity (m = 6.00 cm^2^). In terms of lipids and biomarkers, they had the highest level of HDL cholesterol (m = 60.00 mg/dl), a high percentage of family history of hypercholesterolemia (42.39%) and the best renal function (GFR = 84.88 ml/min/1.73 m^2^). Regarding diabetes-related factors, Cluster 1 members had the lowest values for fasting blood glucose (m = 86.00 mg/dl), diabetes diagnosis (1.10%) and HOMA-IR (m = 1.24). Oppositely, they had the highest insulin sensitivity (Quicki index m = 0.37). Cluster 1 can be considered as the healthiest cluster in terms of cardiovascular health, as they had the lowest values of vascular age (m = 43.00 years), central pulse pressure (m = 38.00 mmHg), pulse wave velocity (m = 7.50 m/s), abnormal ECG reading (10.70%), and systolic blood pressure (m = 120.00 mmHg). Finally, they were also more frequently non-smokers (m = 62.83%), had higher income (3750.00 €/month) and had a higher median time spent sitting (m = 360.00 min/day) and sedentary occupation (m = 59.26%) (Table 1, Fig. 3). The average 10-year cardiovascular risk for Cluster 1 was 0%.

Cluster 2 “Family history—Overweight—High Cholesterol” encompassed N = 508 participants (37.46% of the total population). Members of Cluster 2 were in the vast majority overweight (m = 28.48 kg/m^2^) with low values of HbA1c levels (m = 37.00 mmol/mol). Overall, they had intermediate values for all considered anthropometric features. They were characterized by elevated total (m = 205.00 mg/dl) and LDL cholesterol levels (m = 128.50 mg/dl). They also had a high frequency of family history of diabetes (25.00%) and a high percentage of family history of high blood pressure (43.70%). The average 10-year cardiovascular risk for Cluster 2 was 2%.

Cluster 3 “Severe Obesity—Prediabetes—Inflammation” encompassed N = 91 participants (6.71% of the total population). Cluster 3 included individuals with obesity or severe obesity with a higher BMI (m = 35.69 kg/m^2^) and a higher HbA1c level (m = 39.00 mmol/mol) than those in Cluster 2. Cluster 3 was characterized by the highest values for all considered anthropometric features—except waist-to-hip ratio—with elevated waist circumference (m = 114.00 cm), hip circumference (m = 118.30 cm) or fat mass percentage (m = 42.20%). They had the highest level of inflammation, based on CRP levels (m = 3.03 mg/l). Cluster 3 members had intermediate values for all diabetes-related factors and cardiovascular health factors. There was an over-representation of women in Cluster 3 (61.54%), with a relatively high level of physical activity (3558.00 MET-minutes/week). The average 10-year cardiovascular risk for Cluster 3 was 1%.

Cluster 4 “Diabetes—Hypertension—Poor Cardiovascular Health” encompassed N = 28 participants (2.06% of the population). Members of Cluster 4 were mainly overweight and individuals with obesity (BMI, m = 29.20 kg/m^2^) with elevated HbA1c levels (m = 54.50 mmol/mol). Cluster 4 is characterized by elevated Waist-to-Hip ratio (m = 1.00). Members of Cluster 4 had the highest triglycerides levels of all (m = 149.00 mg/dl). Regarding diabetes-related factors, most of Cluster 4 members had diabetes (89.29%), they had the highest levels of fasting blood glucose (m = 149.50 mg/dl), insulin levels (m = 16.05 μIU/ml) and insulin resistance (HOMA-IR, m = 6.54). Most of them had hypertension (89.29%) and had the highest values for vascular age (m = 61.00 years), central pulse pressure (m = 43.00 mmHg), pulse wave velocity (m = 9.55 m/s), systolic blood pressure (m = 136.50 mmHg) and percentage of abnormal ECG reading (m = 32.14%). When compared to the overall population, Cluster 4 members were the oldest participants (m = 63.24 years) and had elevated time spent sitting (m = 360 min/day) but the lowest frequency of sedentary occupation (m = 46.43%). The average 10-year cardiovascular risk for Cluster 4 was 15%.

## Discussion

In this large, nationwide population-based study, we have observed 4 stable clusters of individuals from the general population with diverse cardiometabolic health profiles. Our study suggests that this classification could help disentangle the heterogeneity in the general population in terms of cardiometabolic health and be used to tailor prevention strategies. Whereas a first group of more than 50% of the total population (Cluster 1 “Healthy”) was characterized with healthy cardiometabolic features and could benefit from a general prevention strategy, the other 3 groups (Clusters 2–4) may benefit from a more personalized and intensive approach to improve their health. Individuals in Cluster 2 “Family history—Overweight—High Cholesterol” may benefit from a more comprehensive strategy regarding overweight/obesity management and cholesterol with a personalized treatment (e.g. through diet, physical activity, psychology or pharmacological treatment) and starting from an early age for individuals with family history of cardiometabolic diseases. This could delay or prevent them from transitioning from Cluster 2 to Clusters 3 or 4^28^. People in the Cluster 3 “Severe Obesity—Prediabetes—Inflammation” may benefit from an intense lifestyle management strategy adapted to individuals with moderate obesity^29,30^, or bariatric surgery for those with severe obesity^31,32^ with a close monitoring of the impact on low-grade inflammation levels and the reverse of prediabetes to a normoglycemic status^33,34^. Cluster 4 ‘Diabetes—Hypertension—Poor Cardiovascular Health” are often in a multimorbid state, with diabetes and hypertension simultaneously and for a third of them with an abnormal ECG reading or elevated triglyceride levels. Therefore, they could benefit from an intensive combined approach, personalized according to the socioeconomic profile and occupation, with nutritional/dietary^35^ or lifestyle^36^ interventions, smoking cessation^37^, medication or surgery strategies, targeting both high blood pressure and diabetes with the ultimate objective to reduce arterial stiffness and prevent the occurrence of cardiovascular disease and improve general health status^38,39^.

Overall, these groups may benefit from more efficient prevention and therapeutic strategies. If externally validated, general practitioners could one day rely on this profiling to have a better picture of a new patient when limited information is available and try to optimize several cardiometabolic parameters simultaneously. Some previous attempts of defining metabotypes^40^, i.e. metabolomic profiles or combinations of specific metabolites used for classification of individuals into groups have been proposed to advance cardiometabolic prevention^41^. These approaches, along with other recent technologies (big data analysis of gut microbiota, integration of real-time data from wearables), are still complex and not yet cost-effective to implement in practice^42^ and our approach could help to fill the gap and help move towards precision cardiometabolic prevention.

These findings are also an opportunity to rethink the strategies that can be offered, for instance to people with obesity^43^, with new models developed according to a more refined definition of the targeted sub-population. Cardiometabolic health relies on complex, intricate, physiological relationships between all the considered parameters in this work. These results imply a move from a “one-size fits-all” vision to a precision cardiometabolic prevention approach to tackle cardiometabolic diseases according to the variety of phenotypes observed in the general population^14^.

### Strengths and limitations

This study has numerous strengths. First, the large population size, combined with a unique set of cardiometabolic features or lifestyle and demographic factors, enabled us to extensively and deeply phenotype the general population in terms of cardiometabolic health. It has been shown that the ORISCAV-LUX 2 population was representative of the Luxembourgish adult population in terms of geographical district, but not with respect to sex and age distribution, young and elderly individuals being slightly under-represented and women over-represented. Nonetheless, it has been demonstrated that ORISCAV-LUX 2 is a reliable tool for epidemiological research and for cardiometabolic health monitoring in the adult residents in Luxembourg^20^. We also used a semi-supervised clustering approach, guided by two main features for cardiometabolic health, which seems to be more adapted than totally unsupervised clustering to the reality of the knowledge of cardiometabolic health^13^.

This study also has some limitations. Cluster labelling is always subject to interpretation. We used, to the best of our ability, a systematic approach and relied on the most distinctive characteristics in each cluster to label them. Changing the choice of the key factors to guide the semi-supervised clustering (here BMI and HbA1c) could yield to different distributions, but they were chosen as they are frequently assessed in large populations and valid surrogate of the overall cardiometabolic health status^15–18^. The relatively low number of individuals in clusters 3 and 4 could limit the inference that can be made out of these groups.

Stability of the clusters has been evaluated internally but now there is a need to replicate this approach externally, in other large nationwide population-based studies to evaluate external validation of this grouping. Some factors used to describe the clusters, such as physical activity, are self-reported, and therefore could be reported differently in the clusters. Besides, no mental health nor sleep-related factors were included in the descriptive analysis. In future replication studies, wearable devices could be used to collect objective measures of physical activity and sleep quality, which may be valuable information to add in the cluster description.

## Conclusion

In conclusion, our work provides an in-depth characterization and thus, a better understanding of the general population in terms of cardiometabolic health. Our data suggest that such a clustering approach could now be used to define more targeted and tailored strategies for the prevention of cardiometabolic diseases at a population level. This study provides a first step towards precision cardiometabolic prevention and should be replicated in other contexts. Further studies evaluating the associations between these clusters and subsequent incidence of various cardiometabolic and cardiovascular diseases are warranted.
